# Myocardial Infarct Size and Mortality Depend on the Time of Day—A Large Multicenter Study

**DOI:** 10.1371/journal.pone.0119157

**Published:** 2015-03-11

**Authors:** Stephane Fournier, Patrick Taffé, Dragana Radovanovic, Erik Von Elm, Beata Morawiec, Jean-Christophe Stauffer, Paul Erne, Ahmed Beggah, Pierre Monney, Patrizio Pascale, Juan-Fernando Iglesias, Eric Eeckhout, Olivier Muller

**Affiliations:** 1 Department of Cardiology, University Hospital Center (CHUV), Lausanne, Switzerland; 2 Institute for Social and Preventive Medicine, Lausanne, Switzerland; 3 AMIS Plus Data Center, Institute of Social and Preventive Medicine, University of Zurich, Zurich, Switzerland; 4 Department of Cardiology, Fribourg Hospital, Fribourg, Switzerland; Azienda Ospedaliero-Universitaria Careggi, ITALY

## Abstract

**Background:**

Different studies have shown circadian variation of ischemic burden among patients with ST-Elevation Myocardial Infarction (STEMI), but with controversial results. The aim of this study was to analyze circadian variation of myocardial infarction size and in-hospital mortality in a large multicenter registry.

**Methods:**

This retrospective, registry-based study was based on data from AMIS Plus, a large multicenter Swiss registry of patients who suffered myocardial infarction between 1999 and 2013. Peak creatine kinase (CK) was used as a proxy measure for myocardial infarction size. Associations between peak CK, in-hospital mortality, and the time of day at symptom onset were modelled using polynomial-harmonic regression methods.

**Results:**

6,223 STEMI patients were admitted to 82 acute-care hospitals in Switzerland and treated with primary angioplasty within six hours of symptom onset. Only the 24-hour harmonic was significantly associated with peak CK (p = 0.0001). The maximum average peak CK value (2,315 U/L) was for patients with symptom onset at 23:00, whereas the minimum average (2,017 U/L) was for onset at 11:00. The amplitude of variation was 298 U/L. In addition, no correlation was observed between ischemic time and circadian peak CK variation. Of the 6,223 patients, 223 (3.58%) died during index hospitalization. Remarkably, only the 24-hour harmonic was significantly associated with in-hospital mortality. The risk of death from STEMI was highest for patients with symptom onset at 00:00 and lowest for those with onset at 12:00.

**Discussion:**

As a part of this first large study of STEMI patients treated with primary angioplasty in Swiss hospitals, investigations confirmed a circadian pattern to both peak CK and in-hospital mortality which were independent of total ischemic time. Accordingly, this study proposes that symptom onset time be incorporated as a prognosis factor in patients with myocardial infarction.

## Introduction

Several clinical studies have reported circadian variation of ischemic burden among patients with acute ST-Elevation Myocardial Infarction (STEMI) [1–3]. Circadian variation was independent of ischemic time (time between symptom onset and revascularization) and supported by experimental animal models of a genetically modified circadian cycle [4]. Reiter et al. [2], and our team [1], found higher peak creatine kinase (CK) activity (as a proxy for myocardial infarction (MI) size) for patients with symptom onset occurring between 00:00 and 05:59. In contrast, Suarez-Barrientos et al. [3] found significantly higher peak CK and peak Troponin I in patients with symptom onset occurring between 06:00 and 11:59. However, this time group had a significantly higher proportion of anterior wall MI (48.7%), and a significantly lower rate of primary percutaneous coronary intervention (77.3%). More recently, these results were challenged by a multicenter, multiethnic study of 1,099 patients in Italy, Scotland, and China, whose authors, Ammirati et al., did not concur with previous conclusions [5]. Nevertheless, different critical aspects, such as the use of an overly simple trigonometric transformation and the potential bias due to the use of multiethnic cohorts when investigating circadian rhythms, have been mentioned [6]. The present study was the first to assess the circadian variation of ischemic burden and in-hospital mortality in a large and well-defined population of patients with acute STEMI who were treated with primary percutaneous coronary intervention (PCI) and whose data were collected in a prospective registry.

## Materials and Methods

### Definitions

STEMI was defined according to the criteria of the Joint ESC/ACCF/AHA/WHF Task Force for the Universal Definition of Myocardial Infarction [7]. Previous MI or angina pectoris and diabetes were considered if the patient had been diagnosed or treated for these conditions previously.

### AMIS PLUS, study population, and data collection

AMIS Plus is a large multicenter registry that has been collecting data on patients with acute coronary syndrome (ACS) in Switzerland since 1997 [8]. To date, 82 acute-care hospitals in Switzerland treating ACS have continuously enrolled patients in AMIS Plus. Briefly, anonymized patient data are centralized at the AMIS Plus Data Center where they are checked for plausibility and consistency and cross-checked when queries arise. The registry currently includes data from over 45,000 patients with ACS, providing information on clinical characteristics as well as diagnostic and therapeutic procedures. Patients are classified on the basis of their definitive diagnosis as having either STEMI, or non-STEMI or unstable angina.

**Fig 1:**
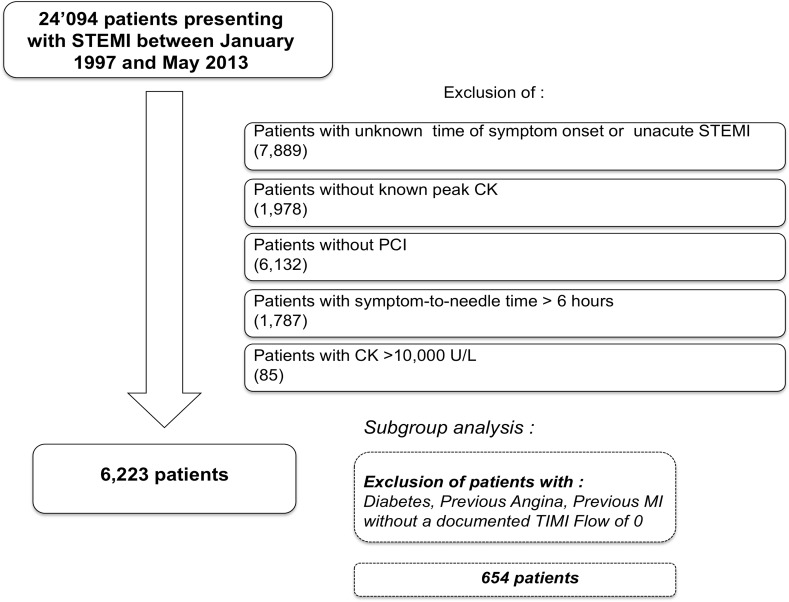
Study flow chart.

For the purpose of this analysis, we selected patients who: (i) had experienced an acute (<12 hours) STEMI between January 1997 and May 2013; (ii) had undergone primary PCI; (iii) had a known time of symptom onset and peak CK values; and (iv) a symptom-to-needle time of less than 6 hours (**Fig. 1**). Furthermore, patients with peak CK > 10,000 were excluded because exceedingly high values are likely due to causes other than STEMI, such as rhabdomyolysis.

### Potential confounders and sensitivity analyses

Potential confounders are variables affecting the relationship between peak CK and STEMI onset time. To assess the impact of potentially confounding variables we performed stratified analyses based on the following dichotomous variables: aspirin intake, age ≤ 85 years, gender, clopidogrel use, anticoagulation treatment, statins use, anterior infarct, moderate to severe renal disease, diabetes, history of MI, previous stable angina, and mean arterial pressure at arrival. We also considered strata defined by ischemic time between [0–2h], [2–4h], and [4–6h], as well as by admission period 1999–2004, 2005–2009, and 2010–2013 (to account for potential trends over time, e.g. new or improved therapeutics or devices). Finally, we investigated the relationship between ischemic time and onset time to assess whether ischemic time might have been longer at night.

### Subgroup analysis

To assess the collective impact of potentially important confounders, we excluded patients with the following criteria: diabetes (known to be associated with silent ischemia); previous angina (potential preconditioning); previous MI (potential modified myocardial mass); and a documented TIMI grade flow > 0 at the start of procedure (**Fig. 1**).

### Statistical analysis

We analyzed the relationship between peak CK and STEMI onset time using polynomial-harmonic regression methods [9]^,^[10] to determine if humans exhibited a circadian dependence of infarct size. The following polynomial-trigonometric regression model $y_{i}=\beta_{0}+\sum_{j=1}^{d}\beta_{j}t^{j}+\sum_{j=1,2,4,8}^{}\left\{\alpha_{j}\cos\left(2\pi tj/24\right)+\gamma_{j}\sin\left(2\pi tj/24\right)\right\}$ was considered and estimated by maximum likelihood. Outcome *y*_*i*_ was peak CK level and the explanatory variable *t* was symptom onset time. We started with a model including four different pairs of harmonics, i.e. sine and cosine terms of 3-, 6-, 12-, and 24-hour periods, and a polynomial of degree 2. This function was very flexible and allowed for a very precise approximation of any non-linear functional relationships. The significance of the polynomial trend, as well as of each pair of harmonics, was assessed using Wald tests, with type one error set to 5%. In this linear model, the coefficient of determination *R* ^2^ was used to quantify the proportion of explained variation (PEV) by the regressors included into the model. We therefore computed the share of each pair of harmonics to the proportion of explained variation by: ${\text{share}}_{24-\mathrm{hour harmonics}}=\left(R_{24-\mathrm{hour harmonics only}}^{2}-R_{\mathrm{no harmonics}}^{2}\right)/\left(R_{\mathrm{all harmonics}}^{2}-R_{\mathrm{no harmonics}}^{2}\right)$ for the pair of 24-hour harmonics, and likewise for the other pairs of harmonics [11]^,^[12].

A backward selection procedure (with a 5% error rate) was used to identify the best fitting reduced model. The same analyses were also performed in the strata defined by the potentially confounding variables.

The relationship between mortality and STEMI onset time was analyzed in the same way as peak CK, except that instead of using the identity link we used the logit link as the dependent variable was dichotomous. Finally, the relationship between ischemic time and onset time was assessed using nonparametric restricted cubic spline methods. All analyses were carried out using STATA 12.1 software.

### Ethics

This study complied with the Declaration of Helsinki regarding investigations on humans and was approved by the University Hospital Center of Lausanne’s Institutional Ethics Committee, Switzerland.

## Results and Discussion

### Patient selection and characteristics

In total, 24,094 patients with STEMI were enrolled in AMIS Plus between January 1997 and May 2013. Time of symptom onset was unknown or longer than 12 hours (non-acute) in 7,889 patients, and peak CK was unknown in 1,978 patients; all these patients were excluded. Of the remaining 14,227 patients, 6,132 did not undergo primary PCI and were excluded. Of the 8,095 remaining patients, we excluded 1,787 patients with a known ischemic time of more than 6 hours and 85 patients with peak CK > 10,000 U/L. Eventually, 6,223 patients were included **(Fig. 1)**. Patient characteristics are summarized in **Table 1**.

**Table 1:** Patient characteristics

Population characteristics	Overall
Age in years (mean +/− SD)	61.55 +/− 12.42
Male gender (n;%)	4,953 (79.59)
BMI (mean +/− SD)	29.62 +/− 4.23
Obesity (n;%)	1067 (19.31)
**Haemodynamic**	
Killip I (n;%)	5,321 (85.81)
Killip II (n;%)	499 (8.05)
Killip III (n;%)	96 (1.55)
Killip IV (n;%)	285 (4.6)
Systolic blood pressure (in mmhg) (mean +/− SD)	131.15 +/− 27.55
Diastolic blood pressure (in mmhg) (mean +/− SD)	78.71 +/− 18.53
Heart rate (per minute) (mean +/− SD)	75.93 +/− 18.25
**Medication**	
aspirin (n;%)	1,627 (27.08)
clopidogrel (n;%)	330 (6.94)
prasugrel (n;%)	10 (0.52)
oral anticoagulation (n;%)	199 (3.34)
beta-blocker (n;%)	1,313 (21.99)
ACE inhibitors (n;%)	822 (13.84)
angiotensin II inhibitors (n;%)	679 (11.95)
anti-calcium (n;%)	549 (9.24)
amiodaron (n;%)	3 (0.35)
nitrates (n;%)	204 (3.43)
digoxin (n;%)	35 (0.59)
diuretics (n;%)	612 (12.26)
statins (n;%)	1,354 (22.57)
**Commorbidities and cardiovascular risk factors**	
Past MI (n;%)	739 (12.55)
Diabetes (n;%)	631 (10.71)
Family history (n;%)	1,470 (33.82)
Hypertension (n;%)	2,966 (49.97)
Dyslipidemia (n;%)	3,044 (53.76)
Smoking (n;%)	2,740 (47.66)
Ischemic Heart Disease (n;%)	820 (24.03)
Previous stable angina (n;%)	743 (14.91)
Heart Failure with NYHA >2 (n;%)	136 (2.31)
Chronic Lung Disease (n;%)	195 (3.31)
Moderate to severe renal disease (n;%)	179 (3.04)
Peripheral vascular disease (stade >2) (n;%)	136 (2.31)
**Ischemic territory involved**	
posterior (n;%)	688 (14.17)
lateral (n;%)	243 (11.34)
inferior (n;%)	2,430 (49.72)
anterior (n;%)	2,781 (44.09)
undertermined (n;%)	103 (2.13)
**Other**	
GP IIb/IIIa antagonists (n;%)	2,993 (48.56)
Creatinin on admission (μmol/l) (mean +/− SD)	86.57 +/− 30.55
**TIMI FLOW of culprit vessel**	
At the start: 0 (n;%)	983 (70.06)
At the start: I (n;%)	156 (11.12)
At the start: II (n;%)	124 (8.84)
At the start: III (n;%)	140 (9.98)
At the end: 0 (n;%)	60 (1.52)
At the end: I (n;%)	32 (0.81)
At the end: II (n;%)	213 (5.39)
At the end: III (n;%)	3,649 (92.29)
**Outcomes**	
Peak Ck in U/L (mean +/− SD)	2,129.48 +/− 1889.75
Alive at the end of hospitalization (n,%)	6,000 (96.42)

### Circadian variation of peak CK

**Fig 2:**
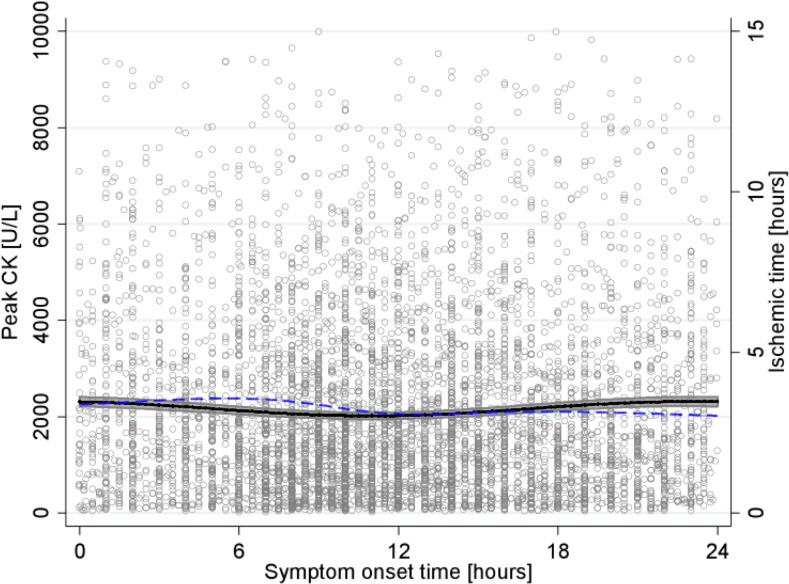
Relationship between peak CK and STEMI onset time. Peak CK level (y-axis on left) in U/L, as a function of symptom onset time, is represented by the solid curve with 95%CI, and ischemic time (y-axis on right) in hours, as a function of symptom onset time, by the dashed curve.

The mean peak CK for the 6,223 patients was 2,129 U/L (standard deviation (SD) 1,890 U/L). When fitting a regression model including the 3-, 6-, 12- and 24-hour period pairs of harmonics as regressors, as well as a polynomial trend, only the 24-hour pair of harmonics was significantly associated with the distribution of peak CK. This suggested a 24-hour circadian rhythm (p = 0.75, p = 0.85, p = 0.06, and p = 0.0002, for the 3-, 6-, 12- and 24-hour period pairs of harmonics, respectively). The overall Wald test for the 3-, 6-, and 12-hour period pairs of harmonics was non-significant (p = 0.28), therefore the backward selection algorithm selected the model including only the 24-hour period pair of harmonics (**Fig. 2, solid curve**). The difference between the minimum and maximum peak CK of the modeled function was 298 U/L, corresponding to an amplitude of 15% (relative to the minimum peak CK value). Accordingly, in the fitted model, maximum peak CK was observed for patients with symptom onset at 23:00 (2,315 U/L), while minimum peak CK was observed for patients with symptom onset at 11:00 (2,017 U/L). The PEV associated with the 3-, 6-, 12-, and 24-hour pairs of harmonics were 3%, 2.6%, 33%, and 73%, respectively. It was, therefore, much larger for the 24-hour pair of harmonics than for the other pairs (which were not statistically significant).

In addition, the relationship between ischemic time and time of symptom onset was plotted **(Fig. 2, dashed curve**). The restricted cubic spline curve showed a maximum ischemic time for patients with symptom onset at 05:30 and a minimum for symptom onset at 00:00. The difference between the modeled minimum and maximum ischemic times was 32 minutes.

### Subgroup analysis

**Fig 3:**
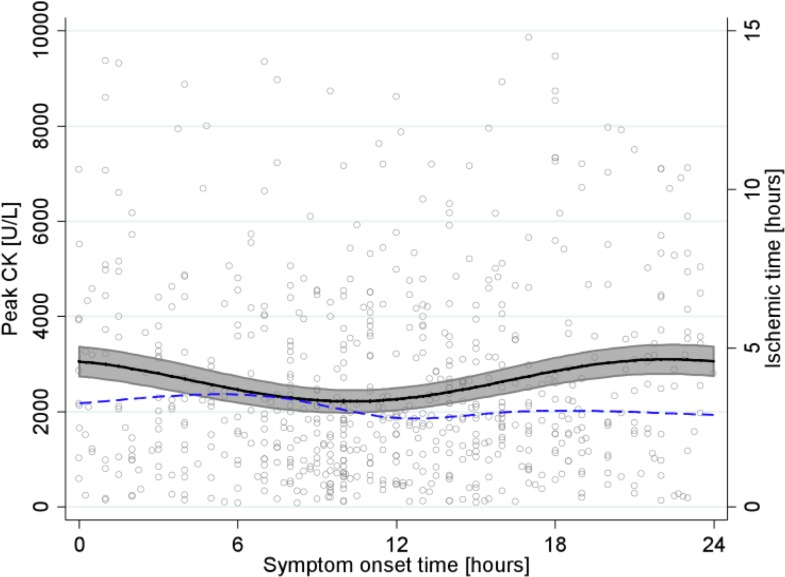
Relationship between peak CK and STEMI onset time in subgroup of patients without diabetes, previous angina, previous MI, and with TIMI grade flow = 0. Peak CK level (y-axis on left) in U/L, as a function of symptom onset time, is represented by the solid curve with 95% CI, and ischemic time (y-axis on right) in hours, as a function of symptom onset time, by the dashed curve.

We identified a subgroup of 654 patients without any history of diabetes, previous angina, previous MI, and with TIMI grade flow = 0 at admission. In these patients the mean peak CK was 2,571 (SD 2,022 U/L). Once again, only the 24-hour pair of harmonics was significantly associated with the distribution of peak CK (p = 0.47, p = 0.07, p = 0.67, and p = 0.002, for the 3-, 6-, 12-, and 24-hour period pairs of harmonics, respectively). The difference between minimum and maximum peak CK of the estimated sinusoidal-cosinusoidal curve was 884 U/L (40% of minimum peak CK). The maximum peak CK (3,099 U/L) occurred for patients with symptom onset at 22:20, while the minimum peak CK (2,215 U/L) occurred for patients with symptom onset at 10:15, thereby suggesting a strong 24-hour circadian rhythm for peak CK variation (**Fig. 3, solid curve**). The PEV associated with the 3-, 6-, 12-, and 24-hour pairs of harmonics were 3%, 6%, 31%, and 64%, respectively. We again plotted ischemic time as a function of the time of symptom onset (**Fig. 3, dashed curve**). There was no relationship between the two curves, and maximum ischemic time was estimated for patients with symptom onset at 04:50.

### Potential confounders and sensitivity analyses

In stratified analyses, we found a consistent circadian variation in each stratum (Figure A to Figure R in S1 File). For example, results in the three sub-periods of 1999–2004, 2005–2009, and 2010–2013 were consistent with the analysis including all 6,223 patients: the amplitudes of the variation in peak CK were 357 U/L, 206 U/L, and 438 U/L, respectively; the times of symptom onset associated with lowest peak CK were 12:00, 11:10, and 10:15, respectively; and the times of symptom onset associated with the highest peak CK were 00:10, 23:10, and 22:15, respectively. Likewise, in strata defined by ischemic time of [0–2h], [2–4h], and [4–6h], the amplitudes of the variation in peak CK were 162 U/L (n = 1032), 299 U/L (n = 3549), and 440 U/L (n = 1642), respectively; times of symptom onset associated with lowest peak CK were 6:50, 11:10, and 11:50, respectively; and the times of symptom onset associated with the highest peak CK were 18:50, 23:05, and 23:59, respectively. In the subgroup of 654 patients with no history of diabetes, previous angina, or previous MI, and with TIMI grade flow = 0 at admission, the amplitudes of the variation in peak CK were 765 U/L, 902 U/L, and 1070 U/L, with the times of symptom onset associated with lowest peak CK at 11:00, 10:15, and 9:00, and the times of symptom onset associated with the highest peak CK at 23:30, 22:20, and 20:40.

### In-hospital mortality

**Fig 4:**
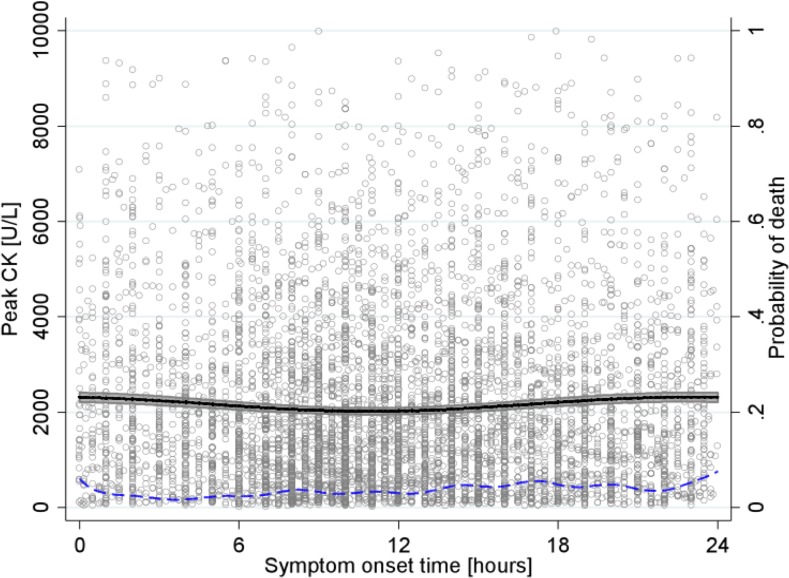
Relationship between peak CK and STEMI onset time. Peak CK level (y-axis on left) in U/L, as a function of symptom onset time, is represented by the solid curve with 95% CI, and probability of death (y-axis on right), as a function of symptom onset time, by the dashed curve.

In-hospital survival data were available for all 6,223 patients: 223 patients died during their hospitalization (3.58%). When using the polynomial-trigonometric regression model, including a polynomial trend and four different pairs of harmonics (**Fig. 4, dashed curve**), the 24-hour harmonic was again—remarkably—the onlyone significantly associated with the probability of death (p = 0.51, p = 0.87, p = 0.11, and p = 0.03, for the 3-, 6-, 12-, and 24-hour period pairs of harmonics). The latter was highest for patients with symptom onset occurring at 00:00 (P(death) = 0.079) and lowest for patients with symptom onset occurring at 03:42 (P(death) = 0.015). **Fig. 4** illustrates that the probability of death is low and varies continuously over the day. Nevertheless, only the 24-hour harmonic was significantly associated with the probability of death.

### Summary of main findings

In a large, national, multicenter registry, we showed that myocardial infarction size and in-hospital mortality followed a circadian rhythm depending upon the time of symptom onset. STEMI patients undergoing PCI, with symptom onset at 23:00, present with the highest peak CK values, while the lowest peak CK values were estimated for patients with symptom onset at 11:00. Importantly, these differences were independent of ischemic time and, secondly, they were even more pronounced in the more homogenous subset of patients with documented TIMI grade flow = 0 at the start of procedure, and no previous MI, diabetes, or angina. The probability of in-hospital death was highest for patients with symptom onset occurring at 00:00.

### Results in context

To the best of our knowledge, this is the largest study to have investigated the circadian variation of peak CK as a measure of ischemic burden among STEMI patients. Its results are in line with earlier findings [1,2]. Using harmonic regression methods to explore the existence of any cyclical patterns, we consistently identified a 24-hour circadian pattern in peak CK and ischemic burden, in various subgroups of individuals, which was independent from ischemic time. It is of note that the same pattern was also observed for mortality, which reinforces our conclusions.

Recent results and conclusions from the multicenter, multiethnic study by Ammirati et al., which included 1,099 patients, are not consistent with ours, however [5]. We believe that their study design may have many sources of potential bias. Firstly, they used a multiethnic and multi-country sample of individuals. Differences in the frequency distribution of clock gene alleles [13], as well as of melatonin levels between ethnicities [14], might have confounded the effect of circadian rhythm and symptom onset time. Indeed, studies with experimental mouse models using clock gene deletion have shown a strong molecular association between time of day and infarct size [4], although the relevance of this finding in humans has not yet been completely established and needs to be confirmed by further studies. Furthermore, melatonin has anti-inflammatory properties and is responsible for lowering blood pressure and normalizing lipid profile. Its level might have been associated with the no-reflow phenomenon. A careful reading of the various figures presented by Ammirati et al. does not completely contradict the observations and conclusions made by both Reiter et al. [2] and our team [1]; in fact, the three figures they gave actually showed the same time trends, but the differences were not statistically significant. As we previously reported [6], the mathematical model used by Ammirati et al. was too simplistic and using a more flexible and appropriate mathematical model [9] is recommended. Finally, the impact of circadian rhythms on cardiovascular physiology is subtle and its effects are moderate. For example, analyses of blood pressure only show a difference of 15% between day and night [15]. Large samples are therefore necessary in order to analyze such effects in the general population.

Different studies have not been able to concur on the precise time of day with the highest vulnerability to myocardial ischemia. Indeed, we previously reported maximum myocardial infarction sizes for patients with symptom onset between 00:00 and 05:59 [1], in line with Reiter et al. who observed maximum myocardial infarction sizes in patients with symptom onset occurring at 01:00 [2]. By analyzing time of symptom onset as a continuous variable with a large sample population, the present study confirmed these findings, although maximum peak CK was observed at 23:00, i.e. earlier than in previous studies. This contrasts with the results by Suarez-Barrientos et al., who found higher peak CK between 06:00 and 11:59 [3]. Nevertheless, even if regional effects such as wake-up-time shift could partially explain these differences, the patients in this time group had a significantly higher incidence of anterior wall MI and a significantly lower rate of primary PCI.

In 2,143 patients with STEMI, Holmes et al. [16] observed a significant association between in-hospital mortality and time at symptom onset: mortality risk ranged from 1.21% at 09:43 to 4.55% at 02:42. However, controlling for heart rate, age, and cardiogenic shock, the association between the time of symptom onset and in-hospital mortality was mitigated and no longer significant. These results were confirmed and pinpointed by the present study by using a larger population: the probability of in-hospital death varied continuously throughout the day, however, it was highest for patients with symptom onset at 00:00. Despite the high fluctuations in the in-hospital death-symptom onset time curve, only the 24-hour harmonic was significantly associated with the probability of death, thereby illustrating the power of polynomial-trigonometric methods in discerning trends in cloudy data. Although no follow-up was available for the present study’s population, we previously reported [1] a significantly higher 30-day mortality in patients with symptom onset occurring between 00:00 and 05:59, and this was in line with these in-hospital observations.

In view of the long period of data collection in our study, several major advances in terms of medication, devices, and even treatment algorithms may have occurred and could have influenced our results. Nevertheless, the three sub-period analyses were consistent with the overall results and, therefore, confirm the robustness of our results.

### Limitations

Our dataset was derived from a registry focusing on MI rather than PCI, and consequently we had no access to data describing procedures. This implies that we could not control for any differences in PCI that might have occurred and could potentially be associated with daytime and outcomes such as mortality.

Nevertheless, data on TIMI grade 3 flow at the end of procedures was available. Furthermore, the usual limitations due to the use of a registry also existed in our study, such as missing data or failure to use the same definitions. Nevertheless, the number of patients in this registry was large enough to detect a significant circadian pattern.

## Conclusions

The present study’s results showed that myocardial infarct size and in-hospital mortality varied according to the time of symptom onset and that this relationship followed a distinctive 24-hour cycle. As this relationship was independent of total ischemic time, the present study supports the notion, derived from experimental data, that the myocardium’s vulnerability to ischemia is subject to a significant circadian pattern. These results suggest that symptom onset time should be considered as a prognostic parameter in STEMI patients undergoing primary PCI less than 6 hours after known symptom onset; symptom onset time should be recorded and analyzed further in future clinical trials dealing myocardial infarction.

## Supporting Information

S1 File Supplementary web figures. Stratified analyses based on aspirin intake, age, gender, clopidogrel intake, anticoagulation regimen, statins use, myocardial infarction location, renal disease, diabetes, hystory of myocardial infarction, previous stable angina, mean arterial blood pressure, admission period 1999–2004, admission period 2005–2009, admission period 2010–2013, ischemic time between [0–2h], ischemic time between [2–4h], ischemic time between [4–6h], respectively. Peak CK level (y-axis on left) in U/L, as a function of symptom onset time, are represented by the green curves for all the patients.
(DOCX)
