# Supplementary material for: Myocardial Infarct Size and Mortality Depend on the Time of Day—A Large Multicenter Study
Source: PLoS One. 2015 Mar 11;10(3):e0119157. doi: 10.1371/journal.pone.0119157 (PMC4356554; doi:10.1371/journal.pone.0119157)
Supplement: S1 File — Stratified analyses based on aspirin intake, age, gender, clopidogrel intake, anticoagulation regimen, statins use, myocardial infarction location, renal disease, diabetes, hystory of myocardial infarction, previous stable angina, mean arterial blood pressure, admission period 1999–2004, admission period 2005–2009, admission period 2010–2013, ischemic time between [0–2h], ischemic time between [2–4h], ischemic time between [4–6h], respectively. Peak CK level (y-axis on left) in U/L, as a function of symptom onset time, are represented by the green curves for all the patients. (DOCX) [file pone.0119157.s001.docx]

**S1 File**

**Figure A**

**Figure B**

**Figure C**

**Figure D**

**Figure E**

**Figure F**

**Figure G**

**Figure H**

**Figure I**

**Figure J**

**Figure K**

**Figure L**

**Figure M**

**Figure N**

**Figure O**

**Figure P**

**Figure Q**

**Figure R**
